# Aspirin Stimulates the Osteogenic Differentiation of Human Adipose Tissue-Derived Stem Cells In Vitro

**DOI:** 10.3390/ijms25147690

**Published:** 2024-07-13

**Authors:** Sarah Funke, Paul Severin Wiggenhauser, Anna Grundmeier, Sara Taha, Benedikt Fuchs, Alexandra Birt, Konstantin Koban, Riccardo E. Giunta, Constanze Kuhlmann

**Affiliations:** Division of Hand Surgery, Plastic Surgery and Aesthetic Surgery, University Hospital, LMU Munich, Ziemssenstraße 5, 80336 Munich, Germany; sarah.funke@campus.lmu.de (S.F.); severin.wiggenhauser@med.uni-muenchen.de (P.S.W.); anna.grundmeier@campus.lmu.de (A.G.); sara.taha@med.uni-muenchen.de (S.T.); benedikt.fuchs@med.uni-muenchen.de (B.F.); alexandra.birt@med.uni-muenchen.de (A.B.); konstantin.koban@med.uni-muenchen.de (K.K.); riccardo.giunta@med.uni-muenchen.de (R.E.G.)

**Keywords:** adipose tissue-derived stem cells, aspirin, osteogenic differentiation, bone tissue engineering, regenerative medicine

## Abstract

This study investigates the impact of acetylsalicylic acid (ASA), also known as aspirin, on adipose tissue-derived stem cells (ASCs), aiming to elucidate its dose-dependent effects on morphology, viability, proliferation, and osteogenic differentiation. Isolated and characterized human ASCs were exposed to 0 µM, 100 µM, 200 µM, 400 µM, 800 µM, 1000 µM, 10,000 µM, and 16,000 µM of ASA in vitro. Cell morphology, viability, and proliferation were evaluated with fluorescent live/dead staining, alamarBlue viability reagent, and CyQUANT^®^ cell proliferation assay, respectively. Osteogenic differentiation under stimulation with 400 µM or 1000 µM of ASA was assessed with alizarin red staining and qPCR of selected osteogenic differentiation markers (RUNX2, SPP1, ALPL, BGLAP) over a 3- and 21-day-period. ASA doses ≤ 1000 µM showed no significant impact on cell viability and proliferation. Live/dead staining revealed a visible reduction in viable cell confluency for ASA concentrations ≥ 1000 µM. Doses of 10,000 µM and 16,000 µM of ASA exhibited a strong cytotoxic and anti-proliferative effect in ASCs. Alizarin red staining revealed enhanced calcium accretion under the influence of ASA, which was macro- and microscopically visible and significant for 1000 µM of ASA (*p* = 0.0092) in quantification if compared to osteogenic differentiation without ASA addition over a 21-day-period. This enhancement correlated with a more pronounced upregulation of osteogenic markers under ASA exposure (ns). Our results indicate a stimulatory effect of 1000 µM of ASA on the osteogenic differentiation of ASCs. Further research is needed to elucidate the precise molecular mechanisms underlying this effect; however, this discovery suggests promising opportunities for enhancing bone tissue engineering with ASCs as cell source.

## 1. Introduction

Adipose tissue-derived stem cells (ASCs) describe an adult stem cell population that can be found in the stem cell niche of adipose tissue [1]. Since their discovery by Zuk. et al. in 2002, ASCs have become a popular cell source in regenerative medicine, particularly for tissue engineering applications [2,3,4]. The utilization of ASCs in tissue-engineered constructs is advantageous owing to their abundance, easy accessibility through liposuction within the adipose tissue, and their autologous nature, addressing immunogenicity concerns associated with allograft-based approaches. Furthermore, ASCs not only possess the intrinsic ability for self-renewal but also demonstrate the potential to differentiate into diverse lineages, including adipogenic, osteogenic, chondrogenic, myogenic, neuronal, cardiomyogenic, and endothelial pathways [2,3]. The extensive clinical potential of employing ASCs in bone tissue engineering to address post-traumatic bone loss, manage delayed or non-union fractures, and facilitate fusion in degenerated joints suggests a capacity to overcome key challenges linked to autologous bone reconstruction, specifically in eliminating donor site morbidity [5]. 

The osteogenic differentiation of ASCs can be accomplished with biochemical induction using growth factors and surface receptors, with physical cues such as rigidity, porosity, and topography, or with biomechanical signals derived from mechanotransduction stimuli from the surrounding extracellular matrix (ECM) [6,7]. In this context, the chemical induction of ASCs toward an osteogenic lineage is typically achieved by exposing them to a differentiation medium containing low concentrations of ascorbic acid, beta-glycerophosphate, and dexamethasone, resulting in an increased level of alkaline phosphatase (ALPL) activity, increased calcium accretion, and the upregulation of bone-specific genetic markers (including ALPL, bone morphogenetic protein 2 (BMP2), runt-related factor-2 (RUNX2), osteopontin (OPN/SPP1)) over the course of two to six weeks [4,8,9]. In addition, other chemical molecules that possess the potential to enhance the osteogenic differentiation of stem cells have been identified in recent years, holding promise for improving stem cell properties and advancing regenerative approaches [9]. 

In this regard, the nonsteroidal anti-inflammatory drug (NSAID) acetylsalicylic acid (ASA), also known as aspirin, has analgesic, antipyretic, anti-inflammatory, and antithrombotic properties, and has shown potential to enhance the osteogenic differentiation capacity of human mesenchymal stem cells (MSCs) [10], including periodontal ligament stem cells (PDLSCs) [11,12], stem cells from exfoliated deciduous teeth (SHED) [13], and dental pulp stem cells (DPSCs) [14,15]. Furthermore, there is evidence from both cellular and animal studies indicating that aspirin has protective effects on bone health by regulating the balance between bone resorption and bone formation at the stem cell level [16]. Additionally, it has been observed to enhance the survival of osteoblast precursor stem cells and promote their differentiation into osteoblasts [17]. Moreover, aspirin exerts inhibitory effects on the nuclear factor κB (NF-κB) pathway, effectively suppressing the differentiation of osteoclasts [16,17,18]. Consistently with these findings, human epidemiological studies indicate a modest beneficial effect of aspirin on bone mineral density in post-menopausal women [19,20,21,22]. 

On a molecular level, aspirin works as an irreversible inhibitor of the cyclooxygenase-1 (COX-1) and as a modulator of the COX-2 enzyme, suppressing the production of prostaglandins and thromboxanes in vivo. This discovery by John R. Vane was later rewarded with a shared Nobel Prize in 1982 for its significant contribution to medicine [23]. The diverse clinical applications of aspirin, beyond its use as an NSAID, including its ability to decrease the risk of cardio- and cerebrovascular events due to its antiplatelet effect, make it one of the most widely used medications globally [24,25,26]. Recent studies further suggest that aspirin holds promise as a chemo-preventive agent for various malignancies, including colorectal, breast, lung, stomach, ovarian, hepatocellular, and prostate cancers [24,25,27]. 

Despite accumulating evidence indicating a stimulatory effect of aspirin on stem cell osteogenesis and its prevalent use among potential stem cell donors, there is, to the best of our knowledge, currently a gap in the literature regarding the impact of aspirin on the stem cell properties of ASCs. Therefore, the primary objective of this study was to investigate the dose-dependent effects of aspirin on the morphology, viability, proliferation, and osteogenic differentiation of ASCs in vitro.

## 2. Results

### 2.1. ASC Characterization

**Figure 1:**
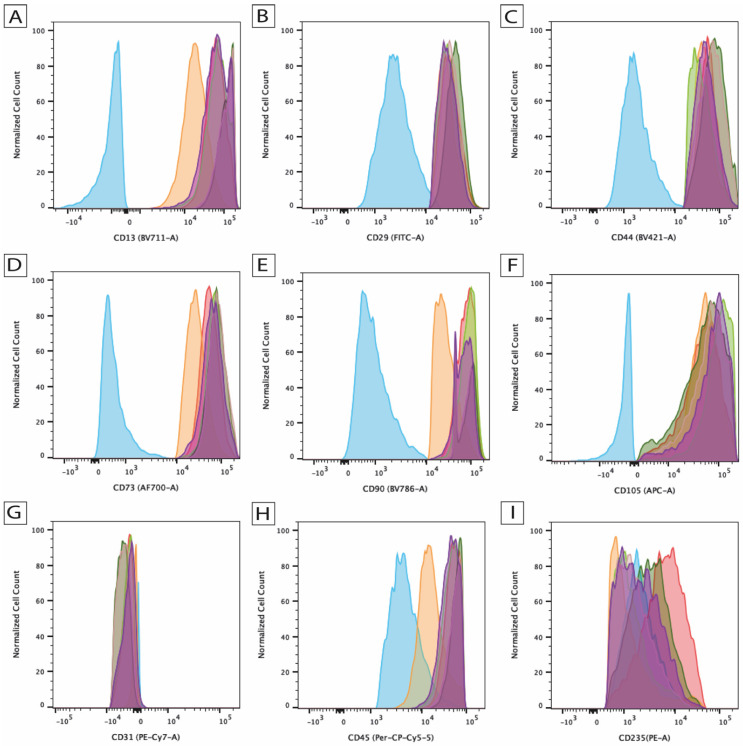
Identification of surface markers on human adipose tissue-derived stem cells (ASCs), including (**A**) CD13, (**B**) CD29, (**C**) CD44, (**D**) CD73, (**E**) CD90, (**F**) CD105, (**G**) CD31, (**H**) CD45, and (**I**) CD235 using flow cytometry. Each histogram contains a negative isotype-specific antibody control (blue) as well as cells from five donors illustrated in different colors (*n* = 5).

To identify the isolated cell population as ASCs, typical surface markers were analyzed by flow cytometry. Based on the joint statement of the International Federation for Adipose Therapeutics and Science (IFATS) and the International Society for Cellular Therapy (ISCT), ASCs are characterized by CD90, CD73, CD105, and CD44 positivity and CD45 and CD31 negativity [28]. These can be detected in all donors. (Figure 1) Furthermore, the trilineage differentiation capacity of the cells and the ability to undergo self-renewal was demonstrated in a previous publication [29]. Consequently, in accordance with the joint statement of the IFATS and ISCT, cells can be characterized as ASCs [28]. 

### 2.2. Mitochondrial Metabolic Activity of ASCs under Dose-Dependent ASA Exposure

**Figure 2:**
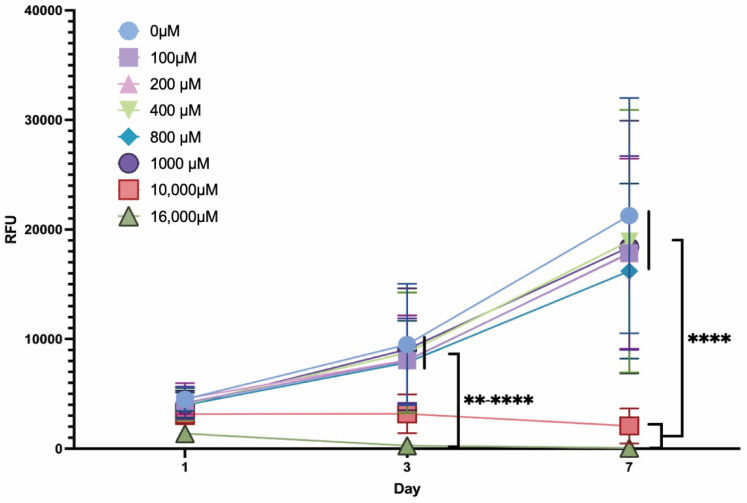
Mitochondrial metabolic activity (viability) of ASCs in the presence of ASA in different concentrations (legend). (*n* = 5; ** *p* < 0.01; **** *p* < 0.0001; mean ± SD).

To evaluate cell viability under the influence of dose-dependent ASA exposure an alamarBlue assay was conducted. The results revealed a significant increase (*p* < 0.0001) in relative fluorescence absorbance units (RFUs) over time for exposure with ASA concentration ≤ 1000 µM with no significant difference to the control group (0 µM of ASA). ASA concentrations > 1000 µM (10,000 µM and 16,000 µM) impaired the mitochondrial metabolic activity, which is equivalent to aerobic cellular respiration, revealing highly significant differences in comparison to the remaining concentrations after 3 (16,000 µM: *p* < 0.0001–0.01) and 7 (10,000 µM and 16,000 µM: *p* < 0.0001) days of cell culture with a visible decrease in their respective RFUs. However, no significant differences in the mitochondrial metabolic activity of ASCs were found between the remaining groups (ASA doses: 100 µM, 200 µM, 400 µM, 800 µM, 1000 µM) and the control (0 µM) after 3 and 7 days in culture (Figure 2). 

### 2.3. ASC Morphology and Viability under Dose-Dependent ASA Exposure

**Figure 3:**
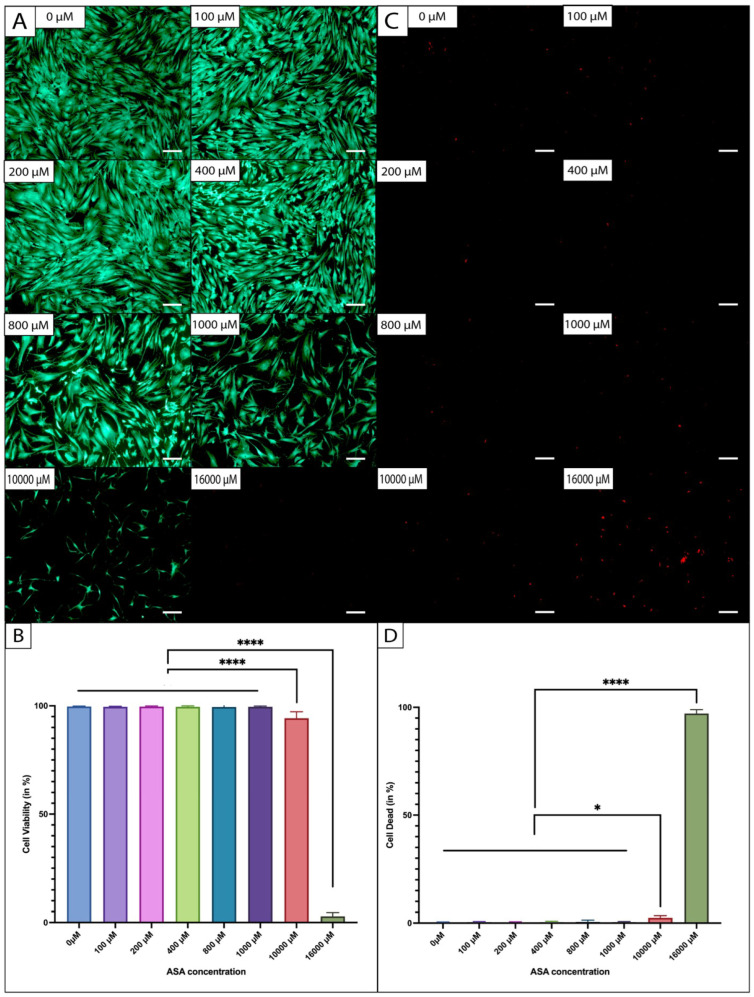
Visual representation of ASCs via live/dead staining in the presence of ASA in different concentrations. (**A**) Fluorescence LIVE staining captured at 5× magnification. (**B**) Relative area of LIVE-stained cells, representing cell viability. (**C**) Fluorescence DEAD staining captured at 5× magnification. (**D**) Relative area of DEAD-stained cells, representing cell death (*n* = 5; * *p* < 0.05, **** *p* < 0.0001; mean ± SD).

Live/dead staining revealed no visible differences in cell morphology when exposed to ASA in lower concentrations in the two-dimensional cell culture after 7 days. ASCs exhibited the typical fibroblast-like phenotype in the presence of ASA in concentrations ≤ 1000 µM (Figure 3A). However, the viability dye indicated a trend towards reduced cell confluency in a monolayer with increasing ASA dosage. Exposure to 16,000 µM of ASA revealed a pronounced cytotoxic effect, with nearly no viable cells and visible dead cells. The quantification of the LIVE fraction (Figure 3B) revealed a highly significant reduction (*p* < 0.0001) in viable ASCs following exposure to doses of 10,000 µM and 16,000 µM of ASA compared to exposure to the groups ≤ 1000 µM of ASA. The quantification of the DEAD fraction (Figure 3C,D) showed significant differences (*p* = 0.0123–0.0327) between 10,000 µM of ASA and the lower-dosed groups. The DEAD fraction after exposure to 16,000 µM of ASA was significantly higher (*p* < 0.0001) compared to the lower-dosed ASA groups. No significant differences were observed between the control group (0 µM of ASA) and ASA exposure with doses between 100 µM and 1000 µM. 

### 2.4. ASC Proliferation under Dose-Dependent ASA Exposure

**Figure 4:**
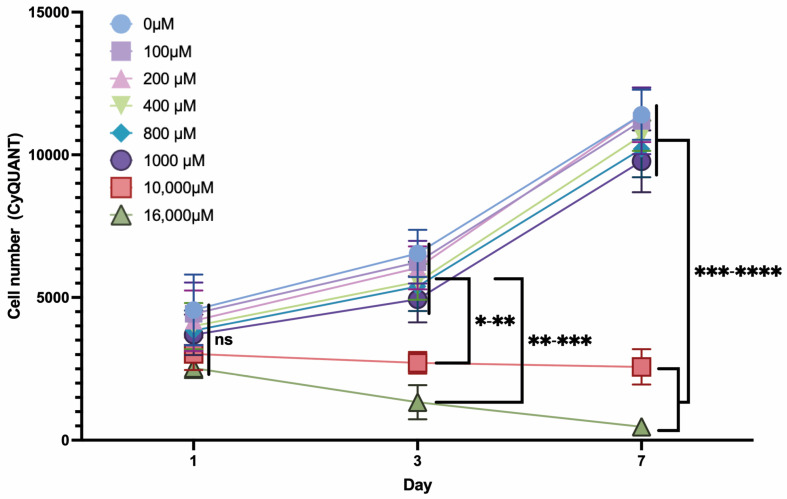
Cell proliferation (CyQUANT^®^ assay) in the presence of ASA in different concentrations (legend) over the course of 7 days. (*n* = 5; ns = not significant, * *p* < 0.05; ** *p* < 0.01; *** *p* < 0.001; **** *p* < 0.0001; mean ± SD).

A CyQUANT^®^ assay was performed to evaluate the total cell number by fluorescence-based measurement of cellular DNA content under ASA exposure (Figure 4). ASA concentrations ≤ 1000 µM showed no significant influence on cell proliferation: the cell number increased significantly (*p* < 0.0001) over time. However, the results indicate a trend towards reduced cell proliferation with increasing ASA dosage ≤ 1000 µM (not significant). ASA concentrations of 10,000 µM and 16,000 µM impeded cell proliferation, leading to a significantly lower cell count after 3 and 7 days of culture in comparison to exposure with lower ASA concentrations. 

### 2.5. Histological Changes during Osteogenic Differentiation of ASCs under ASA Exposure

**Figure 5:**
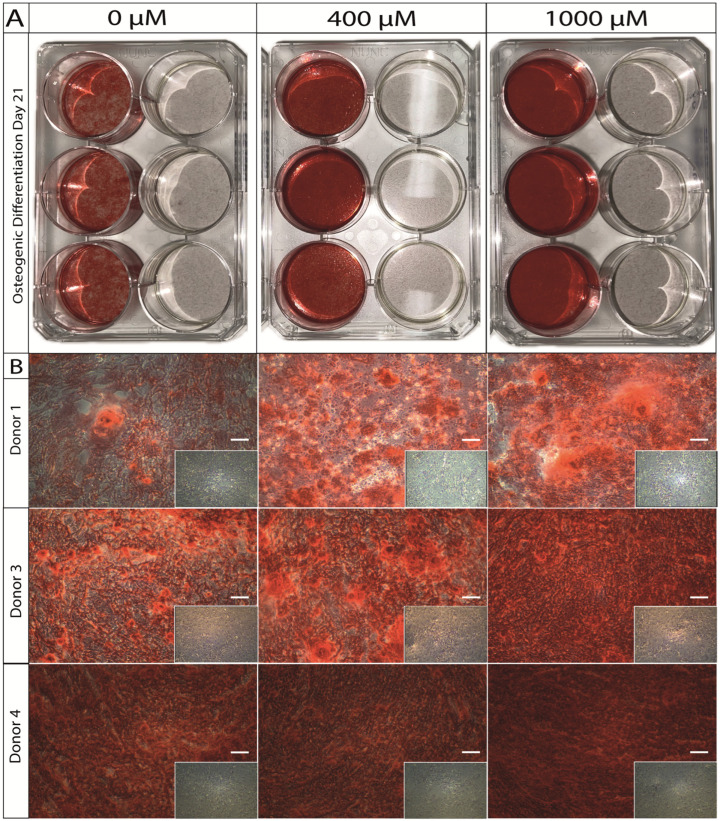
(**A**) Macroscopic picture of the alizarin red staining after osteogenic differentiation over 21 days with 0 µM, 400 µM, or 1000 µM of ASA vs. the respective controls (per 6-well plate: induction, **left**; control, **right**). (**B**) Microscopic (10× magnification) demonstration of donor specific differences after osteogenic differentiation under ASA exposure over 21 days.

**Figure 6:**
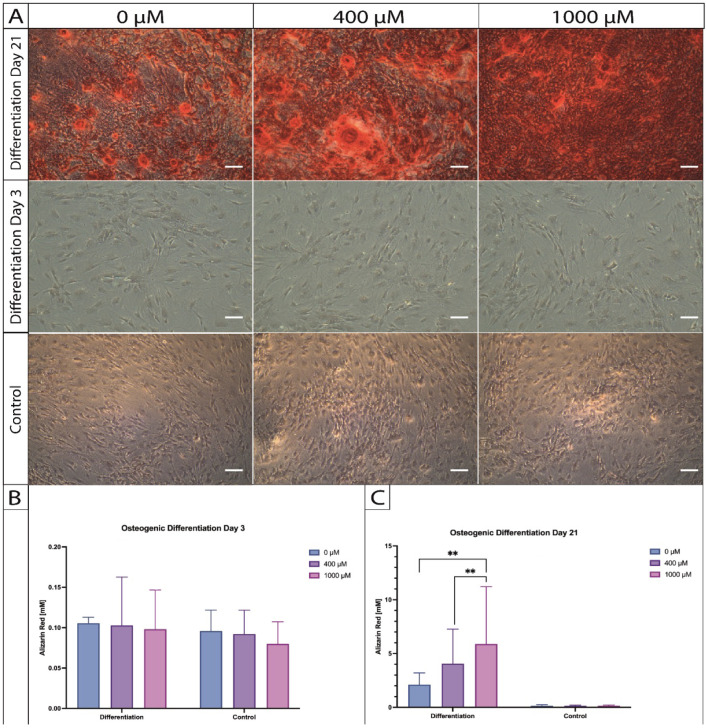
(**A**) Comparison of the different time points (day 3 vs. day 21) of osteogenic differentiation under the influence of ASA (legend) using the alizarin red staining captured at 10× magnification. (**B**) Quantified photometric alizarin red concentrations under ASA influence by day 3. (**C**) Quantified alizarin red concentrations under ASA influence by day 21. (*n* = 5; ** *p* < 0.01; mean ± SD).

An alizarin red staining was performed to visualize calcium depositions during osteogenic differentiation of ASCs under the dose-dependent exposure of ASA over 3 and 21 days. On day 21, a notable difference could be observed macroscopically (exemplary illustration in Figure 5A) and microscopically (exemplary illustrations of 3 donors—Figure 5B) between the different ASA concentrations (0 µM vs. 400 µM vs. 1000 µM) in the induction group. An exposition with 1000 µM showed the strongest uptake of the alizarin red dye. No visual differences could be observed in the control group that received standard culture medium and dose-dependent ASA. On day 3 of osteogenic induction, also no visual differences could be observed between the induction and control group, respectively (Figure 6A). 

The quantification of the alizarin red dye through photometric analysis revealed a significant difference between the samples that received 1000 µM of ASA in comparison to 400 µM (*p* = 0.0099) and to the samples that did not receive ASA (0 µM) (*p* = 0.0092) during 21 days of osteogenic induction (Figure 6C). No significant differences could be found after 3 days of osteogenic differentiation and in the control group (Figure 6B). 

### 2.6. Gene Expression Changes during Osteogenic Differentiation of ASCs under ASA Exposure

**Figure 7:**
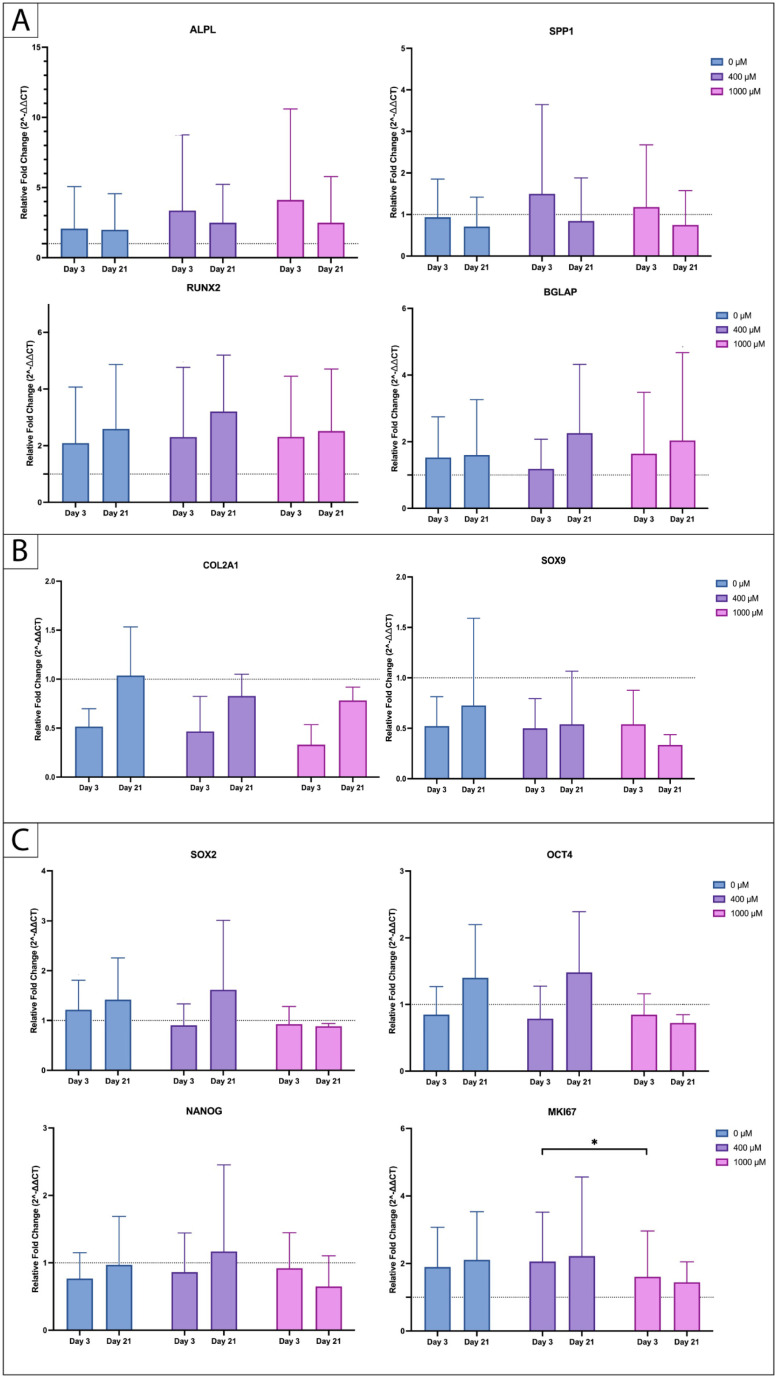
Genetic expression profile of (**A**) early (ALPL, SPP1, RUNX2) and late (BGLAP) osteogenic differentiation, (**B**) chondrogenic differentiation (COL2A1, SOX9), and (**C**) pluripotency markers (SOX2, OCT4, NANOG) and proliferation marker MKI67 under dose-dependent ASA exposure (legend) by day 3 and 21, as measured by qPCR. Relative expressions were normalized to HPRT1 (housekeeping gene). (*n* = 5; * *p* < 0.05; mean ± SD).

To quantify the visible changes at gene expression level, a qPCR was performed. The primary target genes are associated with early osteogenesis, including SPP1, ALPL, and RUNX2, as well as BGLAP, which serves as a marker for late osteogenic differentiation. Additionally, SOX9 and COL2A1 were included as chondrogenic differentiation markers. Furthermore, to provide a more detailed understanding of proliferation and stem cell characteristics, we included stem cell markers SOX2, OCT4, and NANOG and the marker of proliferation MKI67 in the analysis. Our results reveal an upregulation of the osteogenic markers ALPL and RUNX2 during early (day 3) and late (day 21) osteogenic differentiation (Figure 7A). We further observed a trend towards a higher expression of the early osteogenic differentiation markers ALPL and SPP1 after 3 days of osteogenic differentiation, and a more pronounced upregulation of RUNX2 and BGLAP after 21 days under 400 µM and 1000 µM of ASA in comparison to the control (0 µM of ASA), but those results were not statistically significant. The chondrogenic markers (Figure 7B) SOX9 and COL2A1 did not show an upregulation after 3 and 21 days of osteogenic differentiation with or without the ASA exposition. Moreover, the ASA concentrations revealed no effect on the expression of genes related to pluripotency (Figure 7C, SOX2, OCT4, NANOG). We further discovered a significant (*p* = 0.0302) upregulation of the proliferation marker MKI67 in the group that was differentiated under 400 µM of ASA in comparison to the group that was differentiated under 1000 µM on day 3. Compared to the control group (0 µM of ASA), no significant differences regarding MKI67 gene expression were observed. 

## 3. Discussion

ASA, commonly known as aspirin, stands out as one of the most widely used globally medications, due to its multifaceted medical applications ranging from short-term use for pain relief to long-term prevention of thrombotic events. Earlier studies revealed that aspirin has the potential to stimulate the osteogenic differentiation of different stem cell sources, including (murine) bone marrow-derived (bm)MSCs, PDLSCs, SHEDs, and DPSCs [10,11,12,13,30]. However, when compared to other adult stem cell sources, ASCs offer notable advantages. They are easily accessible through liposuction and are almost abundantly available within adipose tissue, yielding sufficient isolates without requiring extensive expansion if obtained from a sizable tissue volume. These characteristics position ASCs as a preferred cell source for applications in regenerative medicine [31,32]. Given the potential impact of ASA on both clinical (e.g., lipofilling, cell-assisted lipotransfer, ASC injection for treatment of joint osteoarthritis [33,34,35,36]) and experimental (e.g., bone tissue engineering [37,38]) applications involving ASCs, this study aimed to explore the dose-dependent effects of ASA on the regenerative capabilities of ASCs, particularly focusing on osteogenic differentiation. 

Based on the previous literature, we selected a concentration range of 100–16,000 µM of ASA for the experiments [10,15,39,40,41]. The oral administration of low-dose aspirin (antiplatelet effect: 75–100 mg) typically yields a peak plasma concentration of 7 µM, while therapeutic analgesic and anti-inflammatory doses (325–3000 mg) result in plasma concentrations typically ranging from 30 to 500 µM [42,43,44]. At 16,000 µM, ASA reaches its maximum soluble concentration. Characterized ASCs were exposed to these different doses of ASA over a seven-day period, revealing no significant impact on the viability and proliferation for ASA doses ≤ 1000 µM in different assays (alamarBlue, live/dead, CyQUANT^®^). However, cell confluency and proliferation appeared to decrease with increasing ASA concentrations ≤ 1000 µM after 3 and 7 days of cell culture, although this trend was not statistically significant. Consistently with these findings, earlier reports involving bmMSCs similarly found no negative impact on metabolic activity with ASA concentrations ≤ 800 µM over 7 days using a CCK8 assay [39]. Additionally, Vukovic et al., who evaluated the effect of lower-dose ASA (10, 50, and 100 µM) on the viability of human DPSCs, did not find a significant difference between the cell viability (MTT-, Neutral Red assay) in the 100 µM of ASA and the control (0 µM) group after 7 days, although a decreasing trend was observed [14]. For exposure to higher ASA doses (10,000 µM and 16,000 µM) our results demonstrated a pronounced cytotoxic and anti-proliferative effect in ASCs as well. Similarly, Hao et al. reported a comparable effect on murine bmMSCs after stimulation with 5000 µM of ASA, and similarly Jiang et al. after stimulation with 3200 µM of ASA [40,45]. Nevertheless, it is worth noting that blood plasma concentrations above 1000 µM are not typically achieved even after the intake of the maximum therapeutic dose of ASA [44]. Due to the cytotoxic effects observed, ASA concentrations above 1000 µM were excluded from the subsequent experiments addressing osteogenic differentiation.

To the best of our knowledge, we are the first to evaluate the effects of ASA on the osteogenic differentiation of ASCs. Our results indicate a stimulatory effect of 1000 µM of ASA on the osteogenic differentiation of ASCs. Alizarin red staining revealed enhanced calcium accretion under the influence of ASA, which was macro- and microscopically visible and highly significant in quantification if compared to osteogenic differentiation without ASA addition over a 21-day period. Notably, stimulation with 400 µM of ASA also exhibited a visible effect on ASC osteogenesis in our experiments. However, the photometric quantification of those results showed a strong trend but no significance in comparison to the induction group without ASA. Previously, Jiang et al. reported comparable results in a study where they incorporated ASA in different concentrations in a synthesized strontium (Sr)-containing α-calcium sulfate hemihydrate/nano-hydroxyapatite composite and evaluated its impact on the osteogenic differentiation of murine bmMSCs in vitro and in vivo. Simultaneously, they also observed more alizarin red-positive cells with an increasing concentration of ASA (max. 800 µM), which was accompanied by an upregulation of osteogenesis-related genes (RUNX2, ALPL, and bone sialoprotein (BSP)) after treatment in osteo-inductive conditions over a one-week period [45]. 

In this context, it is important to understand the process of osteogenic differentiation of MSC sources, including ASCs. They undergo four main differentiation steps (MSC → preosteoblast → immature osteoblast → mature osteoblast), which are primarily subject to regulation by RUNX2 [41,46,47]. RUNX2 serves as a specific regulator in the stem cells’ pathway towards osteogenic differentiation and inhibits it from differentiating toward the adipogenic lineage [48]. More precisely, it initiates the differentiation of MSCs into preosteoblasts and their subsequent transformation into immature osteoblasts and is also expressed in lower concentrations with development into mature osteoblasts [49]. Therefore, we initially focused on the genes SPP1 and ALPL, which are already characteristic of early preosteoblasts and immature osteoblasts. Later, ALPL is also responsible for the regulation of bone mineralization [47]. Once in the immature osteoblast stage, osteopontin (SPP1) is subsequently secreted. After development into mature osteoblasts, osteocalcin (BGLAP) is particularly characteristic [46]. In line with previous publications focusing on other stem cell sources [41,45], our results reveal that osteogenic differentiation was accompanied by a more pronounced upregulation of the early osteogenic differentiation markers (ALPL, SPP1) on day 3 and late osteogenic differentiation (RUNX2, BGLAP) markers on day 21 under ASA exposure (not significant), thus further supporting the hypothesis that ASA concentrations of 400 µM and 1000 µM have osteo-inductive properties in ASCs. Nevertheless, the trend in the genetic expression profile is limited to the small sample size (*n* = 5), and we did not carry out experiments to confirm it on the protein level. 

Considering the underlying molecular mechanism of this phenomenon, it has been previously suggested that the osteo-inductive effect of ASA may be mediated through Telomerase Reverse Transcriptase (TERT), a nucleoprotein responsible for promoting cell proliferation by restoring DNA activity at the telomere’s ends. Liu et al.’s findings indicated that low-dose aspirin enhances TERT activity in SHEDs, thereby increasing their telomere length [13]. This mechanism helps prevent cellular aging during replication and enhances stem cell function, leading to the expression of osteogenic genes during osteoblast differentiation [50]. However, it is noteworthy that this effect was only observed with low-dose ASA (10 and 50 µM), and not in the high-dose ASA group (200 µM), which limits its relevance to our results [13]. Furthermore, previous studies hypothesized that aspirin activates the Wnt7b pathway in osteoblasts, leading to the degradation of phosphorylated beta-catenin. This prevents the differentiation of adipocytes and chondrocytes, consequently enhancing osteoblast activity [41]. Nonetheless, it remains unclear whether these findings are directly applicable to ASCs.

In summary, further studies are required to fully elucidate the underlying molecular mechanism responsible for the osteo-inductive effect of ASA on ASCs. However, the existing knowledge of this effect opens up avenues for enhancing bone tissue engineering in regenerative medicine [41]. One potential approach involves functionalizing scaffold surfaces with a drug delivery system to enable controlled release, improved drug absorption, and more targeted delivery to the desired tissue. For instance, Li et al. previously developed an aspirin-laden liposome delivery system on a polycaprolactone (PCL) scaffold, which effectively promoted osteogenesis of human MSCs both in vitro and in vivo, demonstrating the feasibility and therapeutic potential of such a strategy [51]. Future investigations are needed to adapt and apply this approach to bone tissue engineering using ASCs as the cell source.

While our study provides significant insights into the dose-dependent effects of ASA on the osteogenic differentiation of ASCs, several limitations should be acknowledged. First, the study’s sample size was relatively small (*n* = 5), which may limit the statistical power of our findings. Additionally, our study did not balance female and male donors, which could influence the generalizability of the results. Future studies should aim to include a more balanced and larger cohort of donors to validate and extend our findings.

To translate these findings into clinical practice, further research is essential. This includes determining optimal dosing strategies and conducting comprehensive long-term safety and efficacy evaluations. Clinical trials will be crucial to validate the therapeutic potential of aspirin in bone-related conditions. Furthermore, exploring the incorporation of ASA into scaffold-based delivery systems could offer a promising approach to enhance bone tissue engineering applications. To ensure a robust transition of this experimental approach into clinical settings, in vitro studies followed by in vivo trials in animal models should be prioritized. These steps are necessary to fully understand the mechanisms, optimize the manufacturing protocols, and establish the safety and efficacy of ASA in promoting the osteogenic differentiation of ASCs in experimental bone tissue engineering.

## 4. Materials and Methods

### 4.1. Donor Demographics and Ethics Statement

Adipose tissue was collected from lipoaspirates from five female Caucasian donors (mean age 43.4 ± 13.3 years; mean body mass index (BMI) 35.5 ± 3.21 kg/m^2^) who underwent elective surgery in the department of plastic surgery between April and May 2022. Prior to surgery, explicit written consent was obtained from each donor. Tissue was harvested with waterjet-assisted liposuction (Body Jet evo, human med AG, Schwerin, Germany) from the thighs of the patients. All donors were non-smokers with no significant medical history and were tested negative for HIV, Hepatitis B, and Hepatitis C prior to surgery. Additionally, they reported neither regular nor sporadic use of aspirin. The study was approved by the local ethics committee and was conducted in accordance with the declaration of Helsinki. 

### 4.2. Cell Isolation and In Vitro Culture

The enzymatic isolation of ASCs using a collagen type II (Worthington Biochemical Corp., Lakewood, NJ, USA; Ref: LS004176) solution was performed as previously described by Kuhlmann et al. [29,52]. Cells were cultured in T175 flasks (NuncTM EasYTM Flask 175 cm^2^, Thermo Scientific, Thermo Fisher Scientific, Waltham, MA, USA, Ref: 171658) prepared with 25 mL standard culture medium (88% Dulbecco’s modified eagle medium (DMEM, GibcoTM, Thermo Fisher Scientific, Paisley, Scotland, Ref: 11956-092), 10% fetal bovine serum (FBS, GibcoTM, Thermo Fisher Scientific, Scotland, Ref: 10270-106), 1% Penicillin 10,000 U/mL/Streptomycin 10 mg/mL (PAN-Biotech, Aidenbach, Germany; LOT: 4260422), and 1% Amphotericin B 250 µg/mL (Sigma-Aldrich, St. Louis, MO, USA, Ref: A2942) in a humidified atmosphere (21% O_2_, 5% CO_2_, 37 °C; Heracell Vios 160i, Thermo Scientific, Thermo Fisher Scientific, USA, Serial number: 42231008). Culture medium was changed twice a week until cells reached 80% confluency. Subsequently, cells were cryopreserved for permanent storage in liquid nitrogen using a cryomedium consisting of 10% dimethyl sulfoxide (DMSO, Carl-Roth, Karlsruhe, Germany, Ref: 4720.2) and 90% FBS. The overall cell yield ranged from 7 × 10^6^ to 31 × 10^6^ cells per donor. All experiments were carried out with cells in passage 1.

### 4.3. Fluorescence Activated Cell Sorting (FACS)

FACS analysis was carried out to visualize and characterize the ASC-typical surface markers. This required 106 cells per donor, divided into 0.5 × 10^6^ for antibody staining and 5 × 10^6^ for isotype control. The centrifuged samples were initially incubated for 10 min. at +4 °C with the viability stain, consisting of zombie dye (BioLegend, San Diego, CA, USA, Ref: 423105) diluted at a 1:500 ratio with DPBS (GibcoTM, Thermo Fisher Scientific, Scotland, Ref: 14190-094). Following centrifugation for 5 min. at 500× *g*, the viability stain was removed before proceeding to stain with 100 µL of the antibody staining solution or isotype controls at +4 °C for 30 min. The antibody staining solution comprised 1 µL of each of the nine antibodies examined per sample (Table 1), along with 90 µL of FACS buffer consisting of 1% bovine serum albumin (BSA, Sigma-Aldrich, USA, Ref: A9647-50G) and 1 mM ethylenediaminetetraacetic acid (EDTA, PanReac AppliChem, AppliChem GmbH, Darmstadt, Germany, Ref: A2937) in DPBS. After conducting two washing steps, FACS buffer was added, and the samples were analyzed using a BD LSRFortessaTM Flow Cytometer. Subsequently, data analysis was performed using the FlowJo V10.6.1 software package (both BD Biosciences, Heidelberg, Germany). 

### 4.4. Preparation of the ASA Stock Solution

Following an extensive literature review, a concentration range spanning from 0 µM to 16,000 µM of ASA was established [10,15,39]. The specific concentrations used in the cell viability and proliferation assays were as follows: 0 µM, 100 µM, 200 µM, 400 µM, 800 µM, 1000 µM, 10,000 µM, and 16,000 µM, with 0 µM representing the control group and 16,000 µM representing the maximum soluble concentration of ASA. For the osteogenic differentiation and qPCR, experiments were carried out with 0 µM as the control, along with 400 µM and 1000 µM as defined concentrations of ASA. ASA (>99.0%; Sigma-Aldrich, USA, Ref: A5376) in powder form was weighed under sterile conditions to subsequently dissolve the corresponding amounts of ASA in culture medium.

### 4.5. AlamarBlue Viability Dye

To assess cell viability, a resazurin based alamarBlue assay was performed, using the detection of mitochondrial metabolic activity. In this assay, the dye resazurin is reduced by metabolically active cells to the fluorescent resorufin. The turnover of this dye can be quantified by fluorescence measurement at excitation/emission wavelengths of 560/590 nm. Initially, 25,000 cells per well were seeded in 6-well plates (Corning, New York, NY, USA, Ref: 353224). Subsequently, the standard medium was replaced with the prepared ASA medium, and the cells were further incubated. The Alamar Blue measurements were taken at consistent intervals on day 1, day 3, and day 7, with day 0 marking the onset of ASA exposure. During measurement, ASA medium was replaced with 2 mL of alamarBlue viability dye (10% alamarBlue cell viability reagent (Invitrogen, Thermo Fisher Scientific, Waltham, MA, USA, Ref: DAL1025) and 90% DMEM without phenol red (GibcoTM, Thermo Fisher Scientific, Scotland, Ref: 21063-29)) and incubated for 2 h under standard conditions. For measurement, 100 µL from each well was transferred into a black 96-well plate (NuncTM MicroWellTM 96 Wells, Thermo Scientific, Thermo Fisher Scientific, USA, Ref: 194696) and measured with an Infinite M Plex plate reader (Tecan Trading AG, Männedorf, Switzerland). 

### 4.6. Live/Dead Staining

For visualization of cell viability, ASCs were stained using a live/dead fluorescent staining. Specifically, fluorescein diacetate (FDA, Sigma-Aldrich, USA, Ref: F7378-25G) served as the “live” dye, while cytotoxic propidium iodide (PI, Sigma-Aldrich, USA, Ref: P4864) acted as the “dead” counterpart. The staining procedure followed the experimental setup of the alamarBlue assay, marking the peak of the 7-day growth phase. The live staining solution (FDA) was prepared by dissolving FDA at a concentration of 25 mg/mL in acetone (Acetone 99.7%, Carl-Roth, Germany, Ref: CP40.1), while the dead staining solution was ready to use. ASCs were washed with 1 mL DPBS, incubated with 0.4 µL of FDA in 1 mL DPBS for 5 min at room temperature (RT) in complete darkness, followed by the addition of 20 µL of the dead staining solution and a 5 s incubation at RT. Subsequently, the wells were rinsed three times and bedded in 1 mL DPBS. Images were captured at 526 nm (LDGre) and 613 nm (LDRed) channels at 5× magnification with the Axio Observer KMAT microscope (Zeiss, Oberkochen, Germany). Standardized recordings and uniform post-processing were ensured. The visual representation of live and dead cells was quantified using the open-source software ImageJ 1.53t for Mac (https://imagej.net/ij/ (accessed on 6 July 2024)) using the macro “measure stack” as described previously by Wiggenhauser et al. [53]. The software determined the proportion of colored areas in the total number of pixels and thus provided a relative expression of the areas occupied by cells, respectively. The cell-populated area was determined at a uniformly defined threshold value (20–255) for each dye, and the live and dead-fraction were calculated as percentage (%) of the total cell-occupied areas.

### 4.7. DNA Quantification for Cell Proliferation Analysis

The CyQUANT^®^ Cell Proliferation Assay Kit (Invitrogen™ by Thermo Fisher Scientific, Ref: C7026) was used to quantify cellular DNA content to evaluate cell proliferation under ASA exposure. The assay was performed following the manufacturer’s protocol. For sample preparation, cells were seeded in microplates (96 wells) at a density of 2500 cells per well and cultured according to the previously described experimental protocol. Cell culture medium was discarded, and wells were washed with PBS to minimize the influence of phenol red on subsequent measurements. Following this, plates were frozen at −80 °C to enable effective cell lysis. On the day of the experiment, CyQUANT^®^ GR and dye working solution were prepared: First, the concentrated cell lysis buffer stock solution (20×) was diluted 20-fold in nuclease-free distilled water. After that, CyQUANT^®^ GR dye was diluted 400-fold in the cell lysis buffer working solution (1×) to finalize CyQUANT^®^ GR dye working solution. Next, 200 µL of this solution was added to each well, and the respective plate was incubated for 5 min. at RT and protected from light. Ultimately, fluorescence intensity was measured at excitation/emission wavelengths of 480/520 nm. Cell number was calculated from a calibration curve with predetermined values for each donor.

### 4.8. Osteogenic Differentiation and Quantification

To assess the osteogenic differentiation under ASA influence, ASCs were cultured in six-well plates at a concentration of 50,000 cells per well. Following a five-day incubation in standard medium, osteogenic induction under ASA exposure (with 0 µM, 400 µM, 1000 µM) was induced over a period of 3 or 21 days. The induction group received 98% osteogenic differentiation medium (StemMACs OsteoDiff Media, Miltenyi Biotec, Beach, CA, USA, Ref: 130-091-678), 1% penicillin/streptomycin and 1% amphotericin B) and the control group was cultivated in standard cell culture medium. Alizarin red staining, targeting calcium, followed the manufacturer’s protocol. Briefly, after removing the culture medium, cells were washed with DPBS and fixed with 4% formaldehyde (Microcos GmbH, Rheinfelden, Germany, LOT: 100816) for 15 min. The formaldehyde was removed, and the cells were washed thrice with distilled water. Alizarin red staining (Sigma-Aldrich, USA, ECM815, PartNo. 2003999) was added and incubated for 30 min. at RT. Images were captured using the Axio Observer KMAT (Zeiss, Oberkochen, Germany) at 10× magnification. For the photometric quantification of the ARS concentration, 800 µL of 10% Acetic Acid (Sigma-Aldrich, USA, ECM815, PartNo. 2004807) was added to the well and incubated for 30 min. at RT under slight shaking. Cells were transferred into 1.5 mL Eppendorf tubes using a cell scraper (25 cm/1.8 cm, FalconTM, Corning, USA, Ref: 353086). After incubating the tubes for 10 min. at 85 °C, they were transferred on ice for 5 min. and centrifuged at 20,000× *g* for another 10 min. Subsequently, 800 µL of the supernatant was transferred into a fresh tube, and 300 µL 10% ammonium hydroxide (Sigma-Aldrich, USA, ECM815, PartNo. 2004809) was added to equalize pH levels. OD values were measured at ʎ = 405 nm, and ARS concentration was calculated using a standard curve. 

### 4.9. RNA Isolation, cDNA Synthesis, and Quantitative Polymerase Chain Reaction

For RNA isolation, cells were rinsed with DPBS, trypsinized (TrypLe, Gibco, Ref: 12563-011), and the cell pellet was stored in 1.5 mL Eppendorf Tubes (Eppendorf, Hamburg, Germany, LotNr. L206790P) with 50 µL “RNAlater” RNA stabilization reagent (Qiagen, Hilden, Germany, LotNr: 160027967) at −80 °C. For RNA purification, the RNeasy mini kit (Qiagen, Germany, Ref: 74104) was utilized according to the manufacturer’s instructions. Samples were measured using the Infinite M Plex plate reader and the i-control^TM^ software (Version 1.10, both Tecan Trading AG, Männedorf, Switzerland) at a wavelength of 260 nm (Ratio 280 nm). 

For cDNA synthesis, the transcriptor first strand cDNA synthesis kit (Hoffmann-La Roche, Basel, Switzerland, Ref: 04897030001) was used, following the manufacturer’s protocol. Initially, the Primer Mix (2 µL PCR-Grade Water, 2 µL Random Hexamer Primer, 1 µL oligo (dT)18 Primer) and the Reverse-Transcription Mix (4 µL Transcriptor RT Reaction Buffer, 0,5 µL Protector RNase Inhibitor, 2 µL Desoxynucleotide Mix 1 mM, 0.5 µL Transcriptor Reverse Transcriptase) were prepared. Subsequently, 5 µL of the Primer Mixes was dispensed into the respective PCR reaction tubes (PCR SingleCap 8-piece-SoftStrips 0.2 mL, Biozym Scientific, Hessisch Oldendorf, Germany, LotNr. 21072). Following this, 10 µL of the pre-vortexed RNA samples was pipetted in triplicate. The suspension was then denatured for 10 min. at +65 °C in the thermocycler (Biometra TOne, Analytik-Jena, Jena, Germany) and cooled for 20 min. at +4 °C. Next, 7 µL of the Reverse-Transcription Mix was added, and the samples were incubated according to the following program in the thermocycler: 10 min at 25 °C, 60 min. at 50 °C, 5 min. at 85 °C and 30 min. at 4 °C. For further use, the samples were stored at −20 °C.

Finally, a quantitative polymerase chain reaction (qPCR) was carried out to detect the genes in question. An overview of the primers used is given in Table 2. A master mix consisting of 10 µL InnuMix qPCR MasterMix Probe (Analytik Jena, Germany, Ref: 845-AS-1201000), 1 µL forward primer, 1 µL reverse primer and 3 µL DEPC-treated, DNase- and RNase-free water (Carl Roth, Germany, CasNo. 7732-18-5) was first prepared. This solution was pipetted onto a PCR plate (VWR^®^ PCR Plate, 96-Well, ABI type, VWR, Avantor, Radnor, PA, USA, Ref: 211-0317) with 5 µL of each of the corresponding cDNA samples, and the PCR was started in the qTower3 G touch (Analytik Jena, Germany) following the settings summarized in Table 3. To evaluate gene expression, all samples were normalized against the housekeeping gene (HKG) HPRT1 (ΔCt = Ct_target gene_ − Ct_HKG_), whereby the Ct values (cycle threshold) correspond to the PCR cycles required for a constant fluorescence level. The relative amount of gene expression can be represented as follows: Fold gene expression = 2^−(∆∆Ct)^. Here, the ∆∆Ct value is obtained by subtracting the ∆Ct of the corresponding control group (∆∆Ct = ∆Ct_ASS_treatment_ − ∆Ct_control_). For each concentration (0 µM, 400 µM, 1000 µM), ∆∆Ct values were determined separately by subtracting the average ∆Ct value of the non-differentiated control group from the respective differentiated samples within the same treatment condition. The experiment was carried out in biological duplicate and technical triplicate.

### 4.10. Statistical Evaluation and Data Illustration

All experiments were carried out in triplicate unless otherwise stated. Statistical analysis was performed using GraphPad Prism v10 for Mac (GraphPad Software, San Diego, CA, USA). Gaussian distribution was evaluated using a Shapiro–Wilk test. Depending on the distribution, either an unpaired *t*-test (student’s test) or a Mann–Whitney-U test was used for statistical analysis. For evaluation of two or more groups, we used a two-way analysis of variance (ANOVA) approach with Bonferroni’s multiple comparisons test for post hoc analyses if results were significant. Results are presented as the mean ± standard deviation (SD), and a *p*-value of <0.05 was regarded as statistically significant. The figures were designed with Adobe Illustrator 2023 (Adobe Creative Cloud, Adobe Inc., San Jose, CA, USA). 

## 5. Conclusions

Our study sheds light on the potential of aspirin (ASA) to influence the osteogenic differentiation of ASCs. We observed a dose-dependent effect of ASA on ASCs, with concentrations of 1000 µM showing the most significant stimulation of osteogenesis. These findings suggest that ASA could be a valuable adjunct in bone tissue engineering applications involving ASCs. However, further research is needed to elucidate the precise molecular mechanisms underlying this effect and to confirm its translational applicability for regenerative medicine.

## Figures and Tables

**Table 1:** Overview of the antibodies and isotypes for FACS analysis.

Antibody	Conjugate	Isotype	Manufacturer
CD44	Pacific Blue	Mouse IgG1, k	BioLegend, USA
CD29	FITC	Mouse IgG, k	EBioScience (San Diego, CA, USA), Thermo Fisher Scientific, USA
CD13	BV711	Mouse IgG, k	BioLegend, USA
CD73	AF700	Rat IgG, k	BioLegend, USA
CD90	BV786	Mouse IgG, k	BioLegend, USA
CD105	APC	Mouse IgG, k	BioLegend, USA
CD31	PE-Cy7	Mouse IgG, k	BioLegend, USA
CD45	PerCP	Mouse IgG, k	BioLegend, USA
CD235a	PE	Mouse IgG, k	BioLegend, USA

**Table 2:** Primer sequences and probe design of the qPCR.

Gene	Full Name	Forward Primer Sequence (5′ → 3′)	Reverse Primer Sequence (5′ → 3′)	Length	NCBI RefSeq
*ALPL*	Alkaline Phosphatase	CAAGCACTCCCACTTCATC	CGTCACGTTGTTCCTGTTC	2536 bp	NM_000478
*COL2A1*	Collagen type II alpha 1 chain	TCCATTCATCCCACCCTCTC	AGTTTCCTGCCTCTGCCTTG	5059 bp	NM_001844.5
*HPRT1*	Hypoxanthine phosphoribosyltransferase 1	AGATGGTCAAGGTCGCAAG	AAGGGCATATCCTACAACAAAC	1395 bp	NM_000194
*MKI67*	Marker of proliferation Ki-67	AATCACTAAAATGCCCTGCC	CTTCTTTCACACCTACTTTCCC	11636 bp	NM_001145966
*NANOG*	Nanog homebox	TCTCTCCTCTTCCTTCCTCC	AGTTCTGGTCTTCTGTTTCTTG	1395 bp	NM_024865.4
*OCN/BGLAP*	Bone gamma-carboxyglutamate protein	TCACACTCCTCGCCCTATTG	GTCTCTTCACTACCTCGCTG	506 bp	NM_199173
*OPN/SPP1*	Secreted phosphoprotein 1	AAGTAAGTCCAACGAAAGCC	ACCAGTTCATCAGATTCATCAG	1519 bp	NM_000582.3
*OCT4/POU5F1*	POU domain, class 5, transcription factor 1 isoform 2	AAAGAGAAAGCGAACCAGTATC	TACAGAACCACACTCGGAC	1579 bp	NP_001167002.1
*RUNX2*	RUNX Family transcription factor 2	TCTCACTGCCTCTCACTTG	ACACACATCTCCTCCCTTC	5474 bp	NM_001015051.4
*SOX2*	SRY-box transcription factor 2	GCTCGCAGACCTACATGAAC	GGAGGAAGAGGTAACCACAG	2512 bp	NM_003106
*SOX9*	SRY-box transcription factor 9	AGTTTCTTTGTATTCCTCACCC	TCAAAACACACACACACCC	3931 bp	NM_000346.4

**Table 3:** Amplification conditions set on the qTower3 G touch.

Step	Cycles	Profile	Temperature	Retention Time	Goto	Loops
1	1	Initial denaturation	95 °C	02:00	0	0
2	40×	Denaturation	95 °C	00:30	0	0
3		Annealing	60 °C	01:00	0	0
4		Scan	68 °C	00:30	2	39

## Data Availability

Dataset available on request from the authors.
